# Supplementary material for: A transcomplementing gene drive provides a flexible platform for laboratory investigation and potential field deployment
Source: Nat Commun. 2020 Jan 17;11:352. doi: 10.1038/s41467-019-13977-7 (PMC6969112; doi:10.1038/s41467-019-13977-7)
Supplement: Supplementary file 1 — Supplementary Information [file 41467_2019_13977_MOESM1_ESM.pdf]

A transcomplementing gene drive provides a flexible platform for laboratory investigation and potential field deployment

Víctor López Del Amo[1], Alena L. Bishop[1], Héctor M. Sánchez C.[2], Jared B. Bennett[3], Xuechun Feng[1], John M. Marshall[2], Ethan Bier[1], Valentino M. Gantz[1].*

[1] Section of Cell and Developmental Biology, University of California San Diego, La Jolla, CA 92093, USA.

[2] Division of Biostatistics and Epidemiology, School of Public Health, University of California, Berkeley CA 94720, USA.

[3] Biophysics Graduate Group, University of California, Berkeley CA 94720, USA.

*Correspondence: vgantz@ucsd.edu

Keywords: gene drive, tGD, MCR, split gene drive, population modification, population suppression, Drosophila, CRISPR.

Supplementary Figure 1

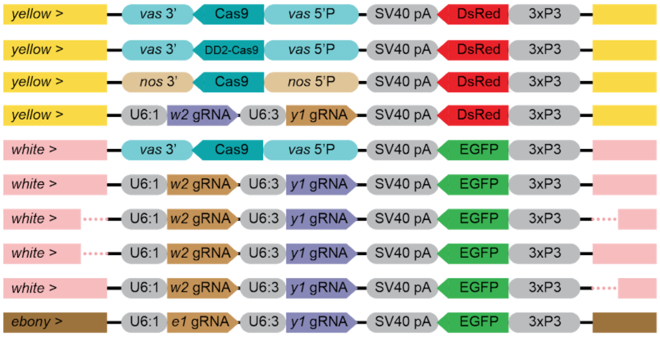

Construct	Locus	Transgene	Used in figures	Generated
pVG182	*yellow*	*vasa*-Cas9	1, 2, 5, Supp. 6	*Lopez Del Amo et al. 2019*[1]
pVG312	*yellow*	*vasa*-DD2-Cas9	Supp. 6	*Lopez Del Amo et al. 2019*[1]
pVG303	*yellow*	*nanos*-Cas9	Supp. 3	This work
pVG186	*yellow*	w2, y1 gRNAs	Supp. 2	This work
pVG316	*white*	*vasa*-Cas9	Supp. 2	This work
pVG185	*white*	w2, y1 gRNAs	2, 5, Supp. 3	This work
pVG307	*white*	Truncated-HAs (both sides)	5	This work
pVMG47	*white*	Truncated-L (PAM-proximal)	5	This work
pVMG48	*white*	Truncated-R (PAM-distal)	5	This work
pVG304	*ebony*	e1, y1 gRNAs	1	This work

Supplementary Figure 1 - Constructs generated for transgenesis. All *vasa* and *nanos* Cas9 lines contain the same *Sp*Cas9 sequence. Two Cas9 lines driven by *vasa* or *nanos* are inserted in *yellow* gene (pVG182 and pVG303 constructs, respectively), and flanked by specific yellow homology arms (yellow boxes) to allow allelic conversion of these transgenes when combined with gRNAs tandem. An additional Cas9 line driven by *vasa* promoter was instead inserted in *white* gene with specific homology arms to allow allelic conversion (construct pVG316). A modified version of pVG182 includes *Escherichia coli* dihydrofolate reductase (ecDHFR) domains that induce Cas9 degradation absence of trimethoprim (TMP). Two different tandem-gRNAs constructs were generated having either the *w2* and *y1* gRNAs or the *e1* and *y1* ones under the control of the U6:1 and U6:3 promoters respectively, inserted at different genomic locations (pVG186 in *yellow*, pVG185, pVG307, pVMG47 and pVMG48 in *white* and pVG304 in *ebony*). Fluorescent markers driven by the eye promoter 3xP3 were used to track each transgene. DsRed was used for transgenes inserted in *yellow* and EGFP was used for constructs targeting *white* or *ebony*. The table on the side summarizes this information including references to what figures in the manuscript use each construct. Constructs pVG128 and pVG312 were previously described[1].

Supplementary Figure 2

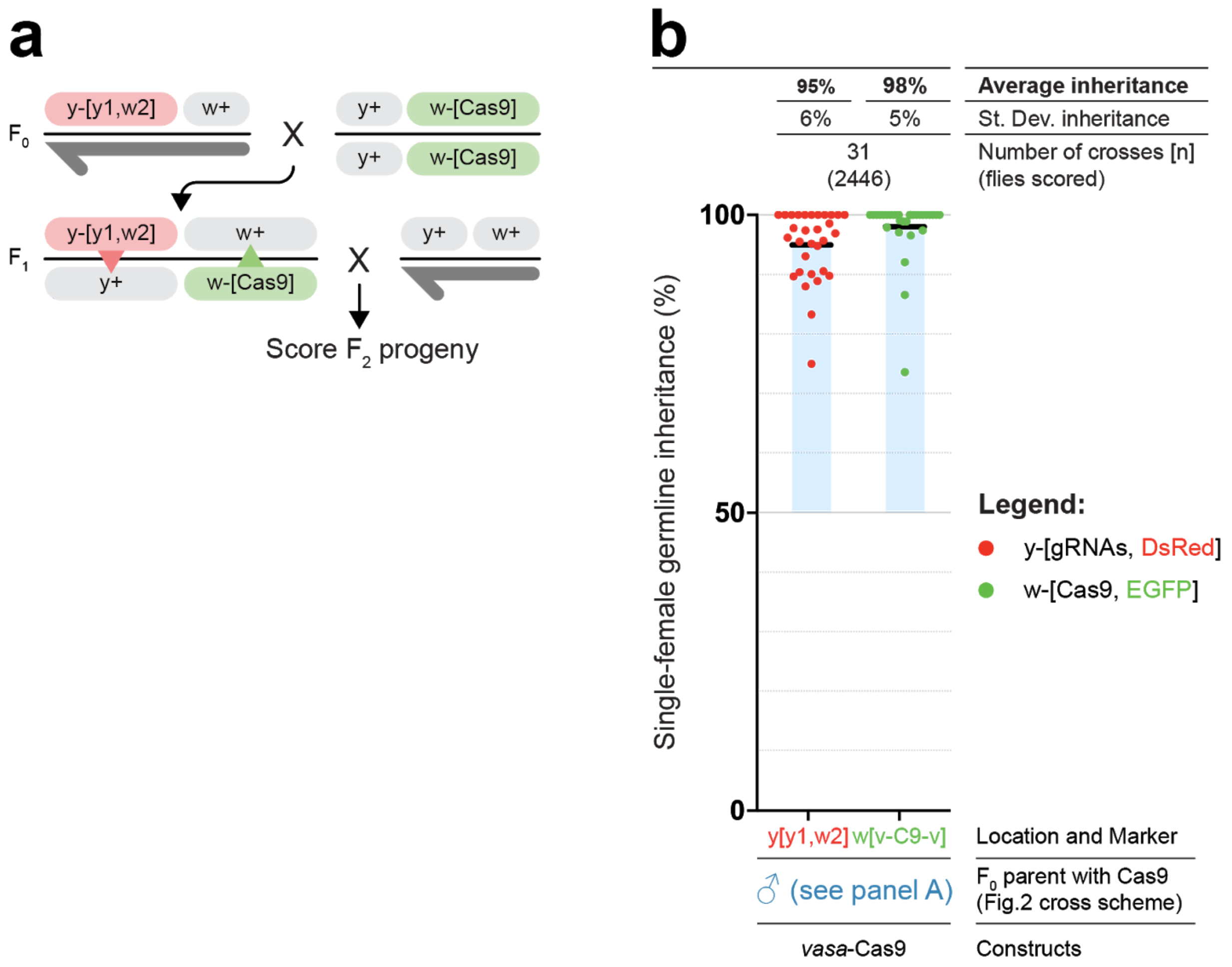

Supplementary Figure 2 - The tGD(w,y) combination alleviates differences observed between Cas9 and gRNA transgenes inheritance observed in the tGD(y,w). The tGD(w,y) "swapped" version has the same Cas9 and gRNAs transgenes than in our tGD(*y,w*), but they are swapped in their insertion locus with the Cas9 inserted in *white* and the gRNAs in *yellow*. (**a**) Shows the genetic cross performed to analyze the F_2 progeny. (**b**) Displays the data points in a plot highlighting the biased inheritance observed. Values for the inheritance average (black bar), standard deviation, number of samples (n) and total number of flies scored in each experiment are represented over the graph in line with the respective data. Raw phenotypical scoring is provided within the Source Data file as "Supplementary Data 2".

Supplementary Figure 3

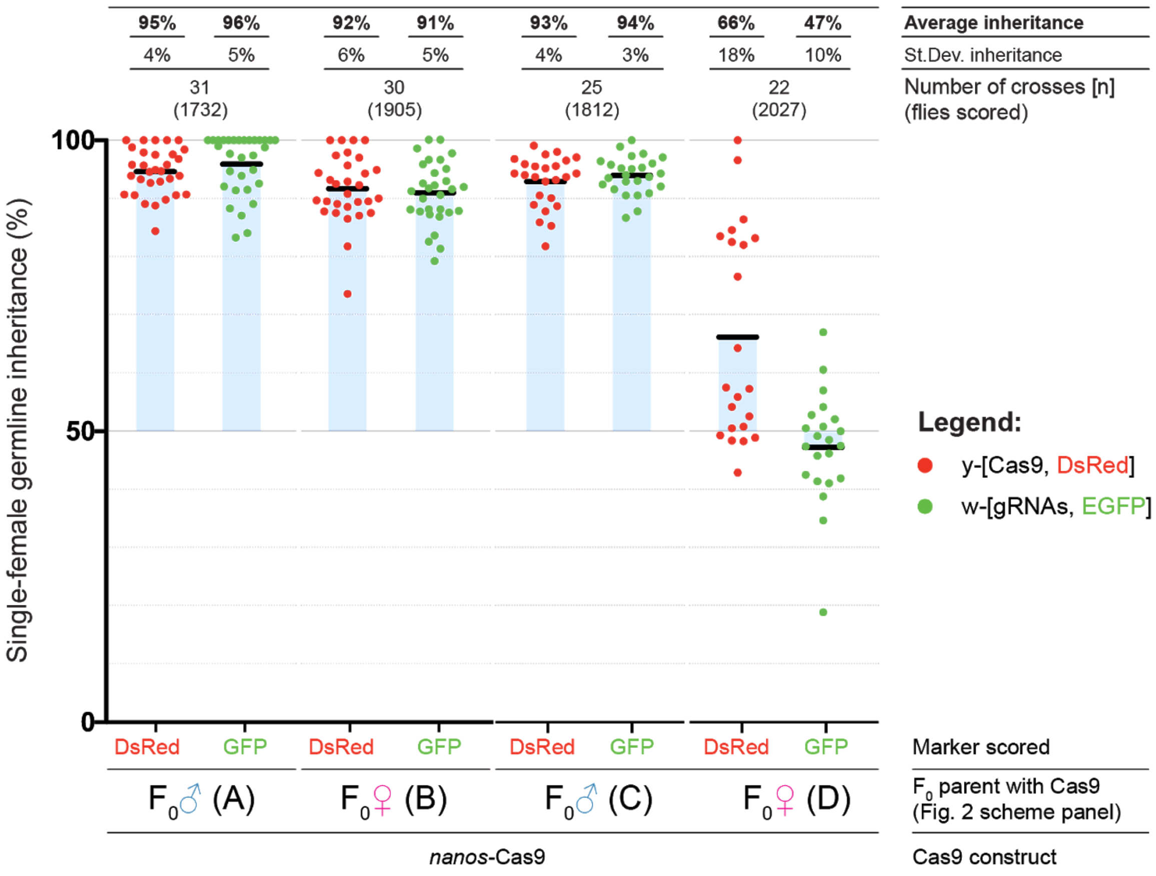

Supplementary Figure 3 - tGD targeting the *yellow* and *white* loci driven by *nanos* promoter. Analysis of the F_2 inheritance rates of the fluorescent markers for the cross scheme combinations from Fig. 2a-d using a Cas9 construct driven by the *nanos* promoter. The inheritance observed in the F_2 progeny of different F_1 females are graphed: (**a**) Cas9 from the F_0 male and gRNAs from the F_0 female, (**b**) Cas9 from the F_0 female and gRNAs from the F_0 male, (**c**) both Cas9 and gRNAs from the F_0 male, and (**d**) both Cas9 and gRNAs from the F_0 female. The blue shading represents the deviation from the expected 50% "Mendelian" inheritance. Values for the inheritance average (black bar), standard deviation, number of samples (n) and total number of flies scored in each experiment are represented over the graph in line with the respective data. Raw phenotypical scoring is provided within the Source Data file as "Supplementary Data 2".

Supplementary Figure 4

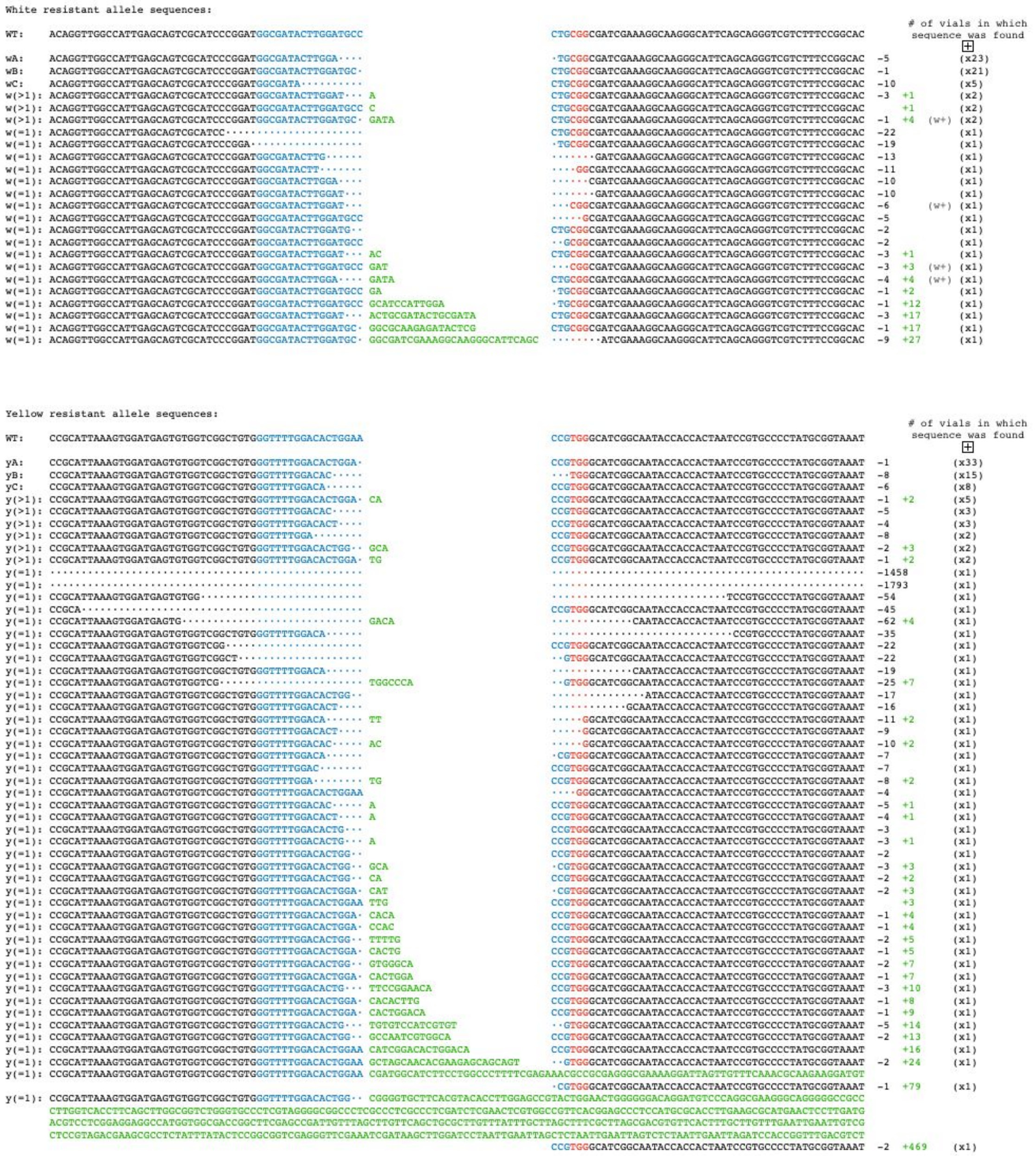

Supplementary Figure 4 - Sequencing of resistant alleles generated in our tGD(y,w) experiments at both *white* and *yellow* loci. These sequences were recovered by sequencing F_2 males recovered from crosses performed in Fig. 2 and Supp. Fig. 3. On top of each list, *white* (top) and *yellow* (bottom), the wild type (WT) sequence is represented. PAM sequence is shown in red, gRNA target sequence in blue, dots represent deleted nucleotides, green letters represent inserted nucleotides. On the left is reported the sequence nomenclature from Fig. 3. On the right, the number of deleted nucleotides in black, followed by number of inserted nucleotides in green, followed by the number of independent vials from which each sequence was recovered. For *white* if the analyzed fly displayed a wild-type phenotype it is followed by a *w+* label.

Supplementary Figure 5

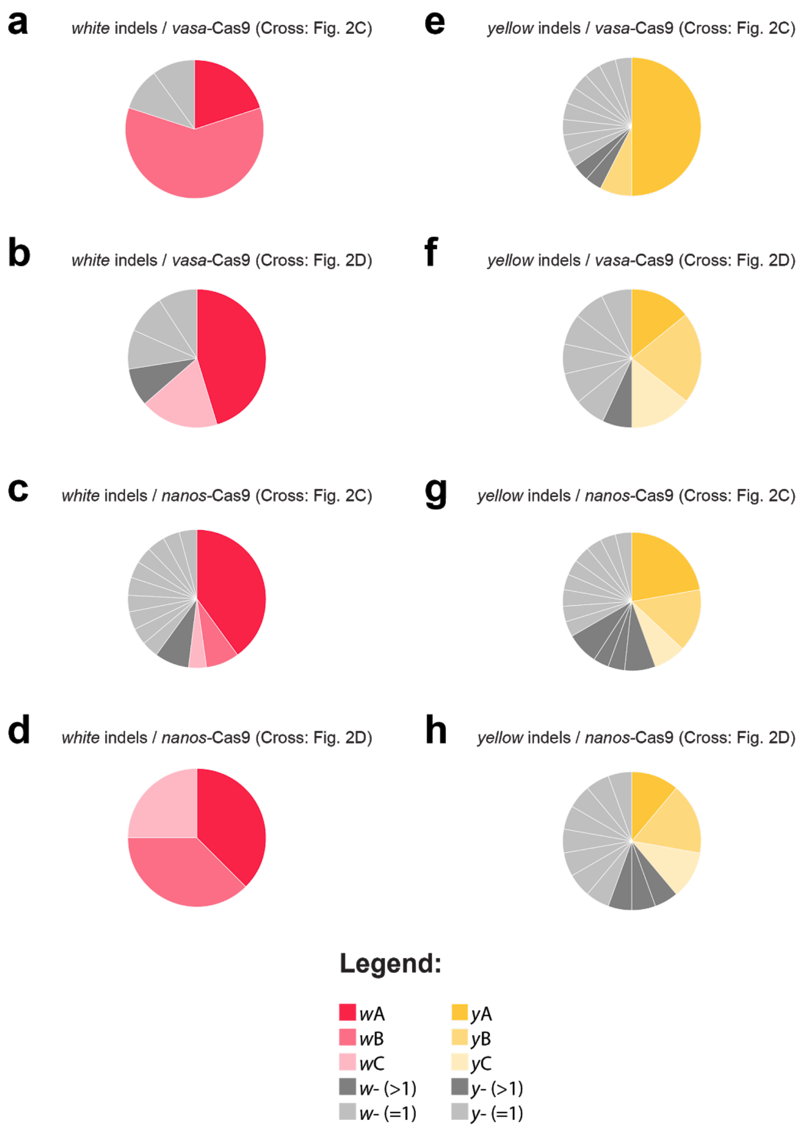

Supplementary Figure 5 - Resistant allele distribution across tGD experiments using different cross schemes. For the cross scheme in Fig. 2C-D we represent in pie charts the distribution of resistant alleles generated at the *white* (**a-d**) and *yellow* (**e-h**) loci when gene drive elements were inherited together and driven by either the *vasa* and *nanos* promoter. Each resistant allele displayed in the pie charts above has independently arisen in different vials. This graphs highlight that specific indel events are favored over others in different conditions. (**a**, **e**) *vasa*-Cas9 and gRNAs inherited from the F_0 male. (**b**, **f**) *vasa*-Cas9 and gRNAs inherited from the F_0 female. (**c**, **g**) *nanos*-Cas9 and gRNAs inherited from the F_0 male. (**d**, **h**) *nanos*-Cas9 and gRNAs inherited from the F_0 female. The color-code legend on the right corresponds to the overall abundance of indel observed in all our experiments, summarized in Fig. 3.

Supplementary Figure 6

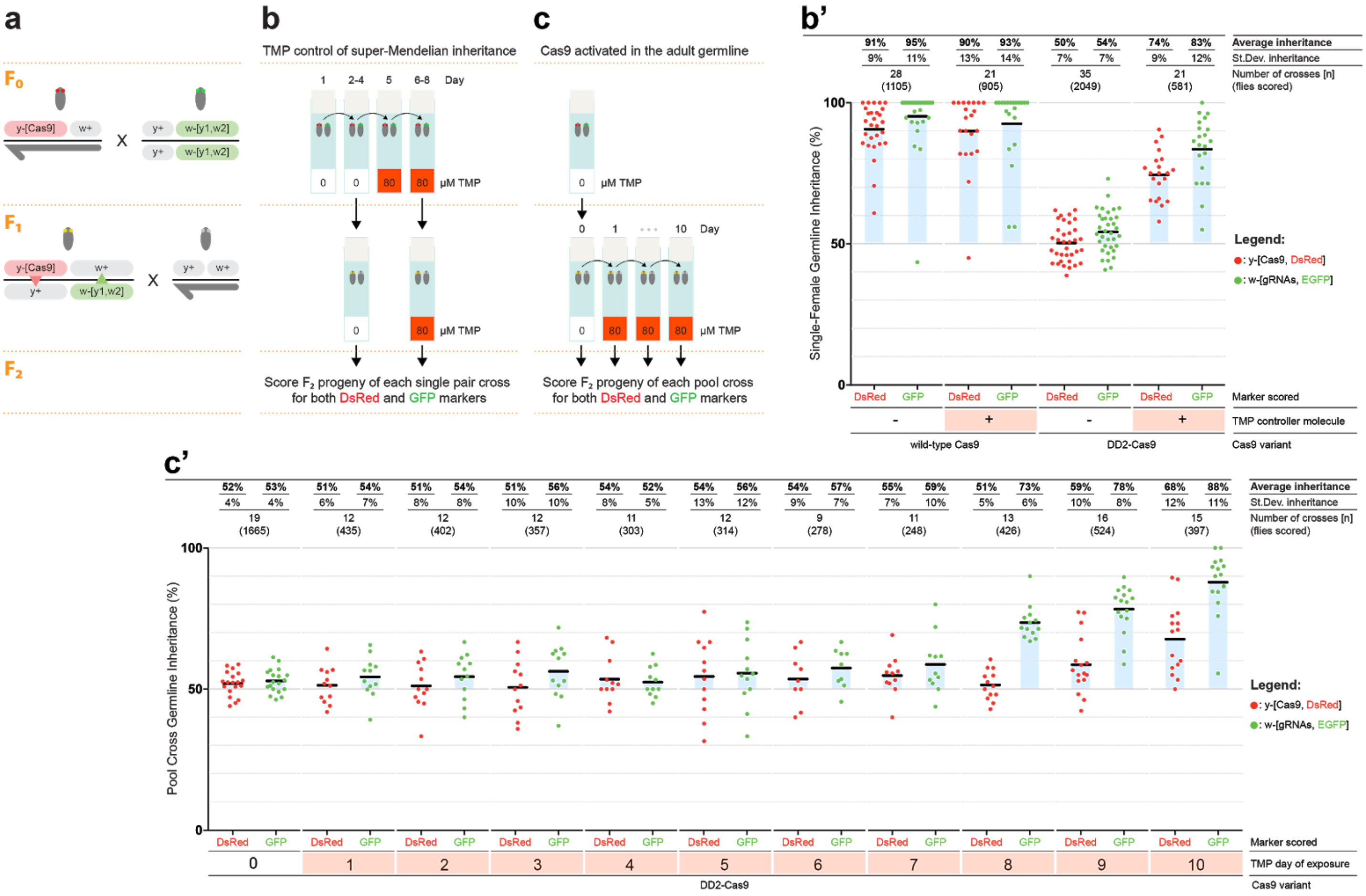

Supplementary Figure 6 - Drug-inducible tGD system allows gene-drive control through a small-molecule feeding. (**a**) Cross strategy performed to test drug-controlled activation of the tGD system. (**b**) Drug feeding strategy to test gene-drive activation during the entirety of fly development. F_0 males carrying either the wild-type Cas9 or the drug-regulated version (DD2-Cas9), both inserted in *yellow* and driven by the *vasa* promoter, were crossed to F_0 females carrying the tandem gRNA transgene in *white*. The F_0 couple was kept on regular food for one day, allowed to lay eggs on a separate vial from day 2–4, kept for one day on a "conditioning" vial containing trimethoprim (TMP), and then passed onto a last vial for egg laying from day 6–8. Emerging F_1 virgin females from the second (day 2–4) and fourth (day 6–8) vials were crossed to wild-type males on the same food condition to analyze the F_1 germline transmission of the two transgenes by scoring the markers in the F_2 progeny. This specific cross scheme allowed us to evaluate the transmission of the fluorescent markers through the germline of F_1 sister females, minimizing potential differences due to genetic background. **(b')** Cas9 crosses are not affected by the presence of TMP, while DD2-Cas9 crosses display Mendelian

inheritance of the markers in absence of TMP and super-Mendelian inheritance when TMP was added to the fly diet. **(c)** Drug feeding scheme to selectively activate allelic conversion in the adult germline. F_0 cross was performed in regular food, and F_1 virgin trans-heterozygous female progeny were collected and crossed first in small pools of 3–4 females to wild-type males on regular fly food. After that, each subsequent day the cross pool was passed onto a new fly tube containing 80 µM of TMP. (**c'**) The F_2 progeny emerging from each tube were analyzed for the inheritance rates of the markers. An average inheritance of ~50% was observed in vials from days 0–7, and an increase in inheritance was seen for days 8–10, suggesting that gametes producing F_2 offspring from days 0–7 had already undergone meiosis at the time of TMP exposure. Raw phenotypical scoring is provided within the Source Data file as “Supplementary Data 3”.

Supplementary Figure 7

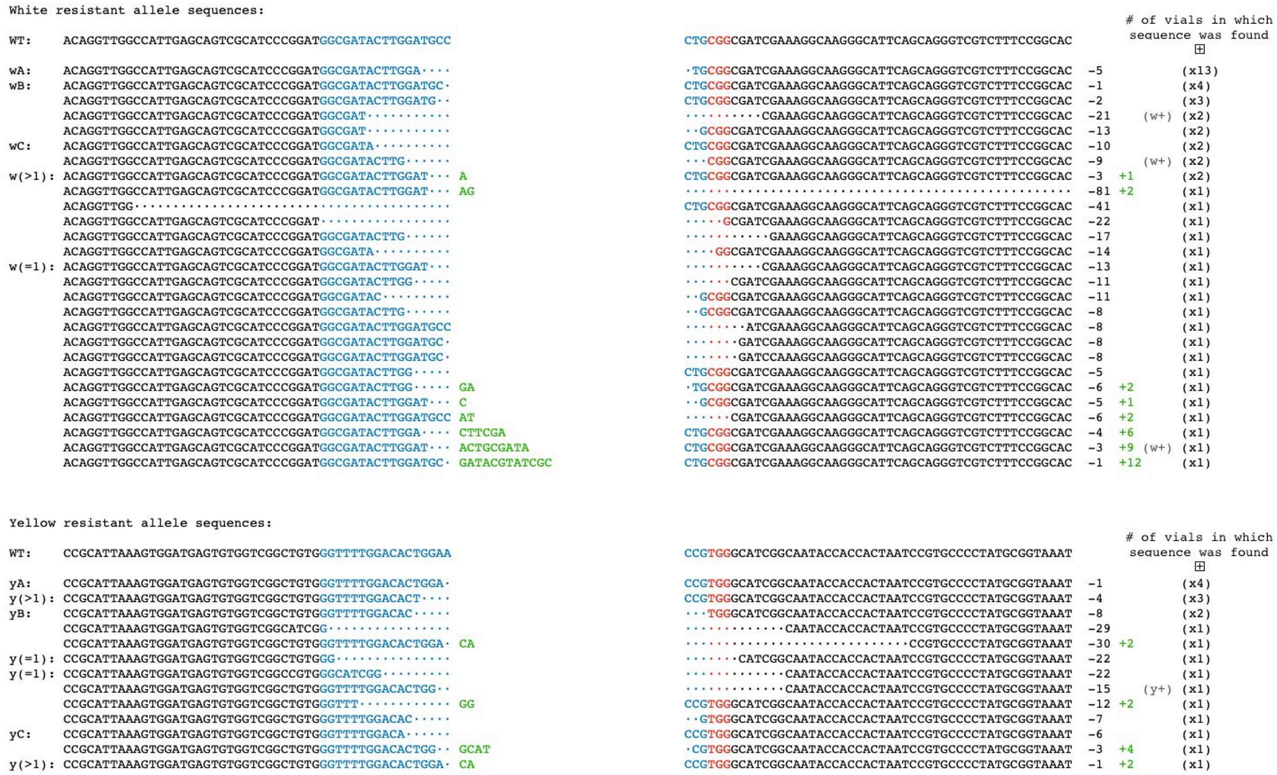

Supplementary Figure 7 - Resistant allele sequences recovered from the drug-mediated activation of the tGD(y,w) in the adult germline. These sequences were recovered by sequencing non-converted F_2 males from Supplementary Fig. 6 representing resistant alleles events generated in the adult germline of F_1 females at the *white* (top) and *yellow* (bottom) loci. On top of each list the wild-type (WT) sequence is represented. PAM sequence is shown in red, gRNA target sequence in blue, dots represent deleted nucleotides, green letters represent inserted nucleotides. On the left is reported the sequence nomenclature from Fig. 3. On the right, the number of deleted nucleotides in black, followed by number of inserted nucleotides in green, followed by the number of independent vials from which each sequence was recovered. If the analyzed fly displayed a wild type phenotype it is marked with either *w+* or *y+*.

Supplementary Figure 8

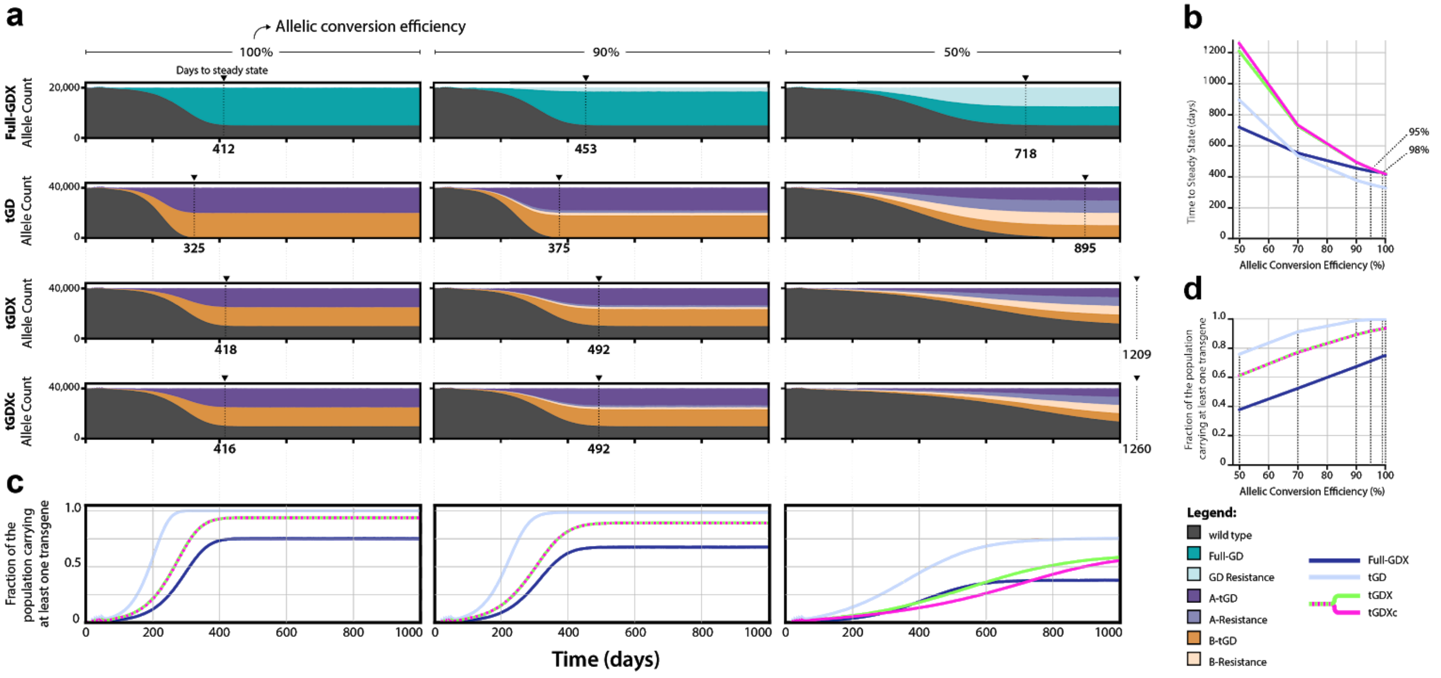

Supplementary Figure 8 - Mathematical modelling of spread of a variety of trans-complementing and full gene drive systems through *Ae. aegypti* populations. Model predictions for releases of *Ae. aegypti* mosquitoes homozygous for the trans-complementing system with: i) components linked on an autosome (tGD), ii) components linked on the X chromosome (tGDX), and iii) components unlinked at two loci on the X chromosome (tGDXc). Also modeled are releases of *Ae. aegypti* mosquitoes homozygous for a full gene drive iv) at an X chromosome locus (Full-GDX). Drive systems are parameterized with ballpark parameter estimates for model exploration: i) a cleavage frequency of 100% in females and males, ii) an allelic conversion efficiency given cleavage of 50-100% in females and males, and iii) no fitness costs associated with the Cas9 or gRNA alleles. All resistant alleles are assumed to be in-frame/cost-free. Five weekly releases are simulated consisting of 100 adult males homozygous for each system into a population having an equilibrium size of 10,000 adults. Model predictions were computed using 50 realizations of the stochastic implementation of the MGDrivE simulation framework[2]. (**a**) Stacked allele counts over time are depicted for the Full-GDX, tGD, tGDX, and tGDXc systems for allelic conversion efficiencies of 100%, 90% and 50%. (**b**) Allelic conversion efficiency plotted against time to steady state for the Full-GDX (dark blue), tGD (light blue), tGDX (green), and tGDXc (pink) systems. Autosomal systems spread faster than X-linked systems due to their ability to drive in both sexes. At high allelic conversion efficiencies (90-100%), autosomal systems spread at similar speeds, as do X-linked systems; however as the allelic conversion efficiency declines (50-90%), the tGD and tGDc systems are slowed down to a greater extent than the Full-GD system (compare to **Fig. 6**). Similarly, the

tGDX and tGDXc systems are slowed down to a greater extent than the Full-GDX system. (**c**) Fraction of the population carrying at least one transgene over time for the Full-GDX (dark blue), tGD (light blue), tGDX (green), and tGDXc (pink) systems for allelic conversion efficiencies of 100%, 90% and 50%. **(D)** Allelic conversion efficiency plotted against fraction of the population carrying at least one transgene for the Full-GDX (dark blue), tGD (light blue), tGDX (green), and tGDXc (pink) systems. For autosomal systems, while resistant alleles accumulate to similar overall proportions for the tGD, tGDc and Full-GD systems, the tGD and tGDc systems are spread across two loci, and so a higher proportion of individuals have at least one copy of a transgene at equilibrium (for allelic conversion efficiencies <100%). Similarly for X-linked systems, the tGDX and tGDXc systems are spread across two loci, and so a higher proportion of individuals have at least one copy of a transgene, as compared to the Full-GDX system (compare to **Fig. 6**).

Supplementary Figure 9

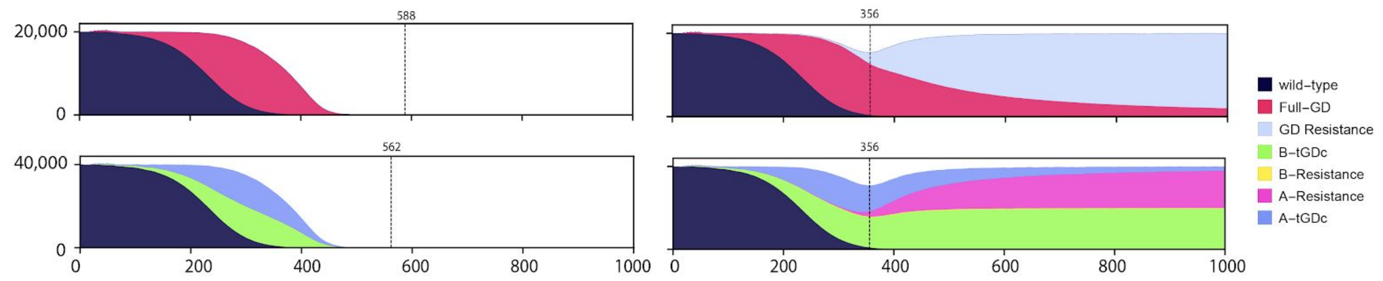

Supplementary Figure 9 - Mathematical modelling of spread of population-suppressing trans-complementing and full gene drive systems through *Ae. aegypti* populations. Model predictions are shown for releases of *Ae. aegypti* mosquitoes homozygous for a full gene drive on an autosome (Full-GD, top), and a trans-complementing system with components linked on an autosome (tGD, bottom). In both cases, the drive targets a gene required in at least one copy for female fertility - for the tGD system, the gRNA locus targets the female fertility gene. Drive systems are parameterized with ballpark parameter estimates for model exploration: i) a cleavage frequency of 100% in females and males, ii) an allelic conversion efficiency given cleavage of 100% (left) and 99% (right) in females and males, and iii) no fitness costs associated with the Cas9 or gRNA alleles. All resistant alleles are assumed to be in-frame/cost-free. Five weekly releases are simulated consisting of 100 adult males homozygous for each system into a population having an equilibrium size of 10,000 adults. Model predictions were computed using 50 realizations of the stochastic implementation of the MGDrivE simulation framework[2]. Stacked allele counts over time are depicted for the Full-GD and tGD systems for allelic conversion efficiencies of 100% (left) and 99% (right). For an allelic conversion frequency of 100%, both the Full-GD and tGD systems induce a population crash within 1.5 years of the final release. For an allelic conversion frequency of 99%, both the Full-GD and tGD systems induce transient population suppression, peaking a population reduction of ~23% at ~1 year after the final release. A resistant allele at the female fertility locus that preserves fertility emerges soon before that time and quickly spreads through the population due to its inherent selective advantage. The tGD allele at the Cas locus does not have a selective disadvantage and remains in the population.

Supplementary Methods

Plasmid Construction: The following tables list the fragments amplified by PCR to perform Gibson assemblies and obtain the constructs indicated above each table. Plasmid sequences are also available on NCBI, and the accession number for each plasmid is provided.

pVG182 - Accession number: MN551085 - (Lopez et al, 2019 BiorXiv)[1]

pVG312 - Accession number: MN551086 - (Lopez et al, 2019 BiorXiv)[1]

pVG303 - Accession number: MN551087

	**Fragment 1**	**Fragment 2**	**Fragment 3**	**Fragment 4**
**Template**	pVG182	Genomic	pVG182	Genomic
**Oligos**	1052-1053	1054-1055	1056-1057	1058-1059

pVG186 - Accession number: MN551088

	**Fragment 1**	**Fragment 2**	**Fragment 3**
**Templat e**	pVG182	pVG182	pVG184_pCFD4-y1-w2 (*)
**Oligos**	621-622	451-623	620-614

* y1 and w2 gRNAs were cloned in pCFD4. pCFD4-U6:1_U6:3tandemgRNAs was a gift from Simon Bullock (Addgene plasmid # 49411 ; http://n2t.net/addgene:49411 ; RRID:Addgene_49411)[3].

pVG316 - Accession number: MN551089

	**Fragment 1**	**Fragment 2**
**Template**	pVG185	pVG182
**Oligos**	611-592	1083-594

pVG185 - Accession number: MN551090

	**Fragment 1**	**Fragment 2**	**Fragment 3**
**Template**	pVG183 (white homology arms in	pVG184	pAAJ120 (EGFP)

	pCR2.1 vector)		
**Oligos**	611-612	614-615	613-451

pVG307 - Accession number: MN551091

	**Fragment 1**	**Fragment 2**
**Template**	pVG185	pVG185
**Oligos**	1069-1070	1067-1068

pVMG47 - Accession number: MN551092

	**Fragment 1**	**Fragment 2**
**Template**	pVG185	pVG185
**Oligos**	612-1070	613-1067

pVMG48 - Accession number: MN551093

	**Fragment 1**	**Fragment 2**
**Template**	pVG185	pVG185
**Oligos**	611-1069	615-1068

pVG304 - Accession number: MN551094

	**Fragment 1**	**Fragment 2**	**Fragment 3**
**Template**	pVG135 (ebony homology arms in pCR2.1 vector)	pVG313_pCFD4-e1-y1 (*)	pVG185
**Oligos**	1075-1076	1077-614	451-1078

* e1 and y1 gRNAs were cloned in pCFD4. pCFD4-U6:1_U6:3tandemgRNA was a gift from Simon Bullock (Addgene plasmid # 49411 ; http://n2t.net/addgene:49411 ; RRID:Addgene_49411)[3].

Oligos used in Gibson assembly cloning:

451	GTACGCGTATCGATAAGCTTTAAGATACATTGATGAGTTTGG
592	CACCTACAACCAGCTGCAGGGATCCACCGGTTAAGATACATTGATGAGTTTGGACAAACC
594	CTTAACCGGTGGATCCCTGCAGCTGGTTGTAGGTGC
611	CTGCGGCGATCGAAAGGCAAGGGCATTCAGC
612	GGCATCCAAGTATCGCCATCCGGGATGCGACTG
613	CGGATGGCGATACTTGGATGCCATATGCGAGCTCGCCCGGGGATCTAATTCAATTAGAG
614	CTTAAAGCTTATCGATACGCGTACGCTAGCACAAAAGCTGGAGCTCCTGCAGG
615	GCCTTTCGATCGCCGCAGACGTCATTTTCAACGTCCTCGATAGTATAGTGG
620	GTGGGTTTTGGACACTGGAAGACGTCATTTTCAACGTCCTCGATAGTATAGTGG
621	TTCCAGTGTCCAAAACCCACAGCCG
622	GAGCTCGGTCGACCGTGGGCATCGGCAATACCACC
623	CCACGGTCGACCGAGCTCGCCCGGGGATCTAATTCAATTAGAGACTAATTCAATTAGAGC
1052	AGATGCCATCGTTCCAGTGTCCAAAACCCACAGCC
1053	AAATCGGCGCGGCACCGGTTAAGATACATTGATGAGTTTGG
1054	TATCTTAACCGGTGCCGCGCCGATTTCAGGGCATCC
1055	CTTATAGTCCATGGCGAAAATCCGGGTCGAAAGTTAC
1056	CGGATTTTCGCCATGGACTATAAGGACCACGACGGAGAC
1057	GGATTCGCCCTCTTTACTTTTTCTTTTTTGCCTGGCCGG
1058	AAGAAAAAGTAAAGAGGGCGAATCCAGCTCTGGAG
1059	TGGACACTGGAACGATGGCATCTTCCTGGCCCTTTTC
1067	ACGTCATTTTCAACGTCCTCGATAGTATAGTGGTTAG
1068	ATATGCGAGCTCGCCCGGGGATC
1069	CGGGCGAGCTCGCATATCGGGATGCGACTGCTCAATGG
1070	GAGGACGTTGAAAATGACGTGGGCATTCAGCAGGGTCGTCTTTC
1075	GTCTCAAGGGTCTGCAGCAAGACTTC
1076	AAAATACCGGTCGATCGACAATTGTGGCAGCG
1077	ACAATTGTCGATCGACCGGTATTTTCAACGTCCTCGATAGTATAGTGGTTAGTATCCC
1078	AGTCTTGCTGCAGACCCTTGAGACGTCCGAGCTCGCCCGGGGATCTAATTCAATTAGAG

Oligos for resistant allele sequencing:

Yellow:

409	CTTTGGCACCTCCACTCCTGCAGGACC
416	CATCAACGATATTTGCGGCCCATTCCAAGC

White:

1127	CTGCTCATTGCACTTATCTACAAG
1129	GCAAATTAAAATGTTACTCGCATCTC

Supplementary References:

1. López Del Amo, V. *et al.* Small-molecule control of super-Mendelian inheritance in gene drives. *Preprint at https://www.biorxiv.org/content/10.1101/665620v1* (2019).
2. C., H. M. S., Sánchez C., H. M., Wu, S. L., Bennett, J. B. & Marshall, J. M. MGDrivE: A modular simulation framework for the spread of gene drives through spatially explicit mosquito populations. *Methods in Ecology and Evolution* **00**, 1–11 (2019).
3. Port, F., Chen, H.-M., Lee, T. & Bullock, S. L. Optimized CRISPR/Cas tools for efficient germline and somatic genome engineering in Drosophila. *Proc. Natl. Acad. Sci. U. S. A.* **111**, E2967–76 (2014).
