# Supplementary material for: A transcomplementing gene drive provides a flexible platform for laboratory investigation and potential field deployment
Source: Nat Commun. 2020 Jan 17;11:352. doi: 10.1038/s41467-019-13977-7 (PMC6969112; doi:10.1038/s41467-019-13977-7)
Supplement: Supplementary file 2 — Description of Additional Supplementary Files [file 41467_2019_13977_MOESM2_ESM.pdf]

Title: Supplementary Data 1 - Phenotypic analysis of the F_2 progeny of tGD(y,e) crosses performed.

Description: Raw counting data of the F_2 progeny phenotypic scoring indicating females and males recovered. Red marker (**DsRed+**), green marker (**GFP+**), both fluorophores (**both**) or no fluorescence (**none**) were scored in order to track Cas9 (red marker) and gRNAs (green marker) transgenes. Transgene inheritance rates in the F_2 progeny for each specific tube (marked as **cross#** in the table) were calculated by combining data from males and females. Average inheritance for both markers and the standard deviation are calculated as well. Data is divided in the following file tabs:

1. **Fig. 1C - tGD(y,e) - Raw data:** tGD targeting *yellow* (Cas9-Red) and *ebony* (gRNA-Green). Inheritance rates using *vasa* promoter with Cas9 inherited from F_0 males.
2. **tGD (y,e)*nanos* - Raw data:** tGD targeting *yellow* (Cas9-Red) and *ebony* (gRNA-Green). Inheritance rates using *nanos* promoter with Cas9 inherited from F_0 males.
3. **Allelic conversion *ebony* transgene, male germline - Raw data:** tGD targeting *yellow* (Cas9-Red) and *ebony* (gRNA-Green). Inheritance rates using *vasa* promoter with Cas9 inherited from F_0 males. In this case, we collected F_1 males to analyze only the allelic conversion in the male germline for our gRNAs tandem inserted in *ebony*.
4. **St: Fig.1B,C vs. ebony transgene, male germline - Statistical Analysis**. Allelic conversion comparison between female and male germline for the Green-gRNAs inserted at the *ebony* locus.

Title: Supplementary Data 2 - Phenotypic analysis of the F_2 progeny of tGD(y,w) crosses performed.

Description: Raw counting data of the F_2 progeny phenotypic scoring indicating females and males recovered. Red marker (**DsRed+**), green marker (**GFP+**), both fluorophores (**both**) or no fluorescence (**none**) were scored in order to track Cas9 (red marker) and gRNAs (green marker) transgenes. Transgene inheritance rates in the F_2 progeny for each specific tube (marked as **cross#** in the table) were calculated by combining data from males and females. Average inheritance for both markers and the standard deviation are calculated as well. The eye color under regular brightfield conditions of each single fly was also scored to distinguish red eye (**w+**), white eye (**w-**) or mosaic eye (**w+/w-**) in both females and males. Data is divided in the following file tabs:

1. **Fig. 2E(A) - tGD(y,w) - Raw data:** tGD targeting *yellow* (Cas9-Red) and *white* (gRNA-Green). Inheritance rates using *vasa* promoter with Cas9 inherited from F_0 males.
2. **Fig. 2E(B) - tGD(y,w) - Raw data:** tGD targeting *yellow* (Cas9-Red) and *white* (gRNA-Green). Inheritance rates using *vasa* promoter with Cas9 inherited from F_0 females.

3. **Fig. 2E(C) - tGD(y,w) - Raw data:** tGD targeting *yellow* (Cas9-Red) and *white* (gRNA-Green). Inheritance rates using *vasa* promoter with Cas9 and gRNA transgenes inherited from F_0 males.
4. **Fig. 2E(D) - tGD(y,w) - Raw data:** tGD targeting *yellow* (Cas9-Red) and *white* (gRNA-Green). Inheritance rates using *vasa* promoter with Cas9 and gRNA transgenes inherited from F_0 females.
5. **Supp. Fig. 2 - tGD(w,y) - Raw data:** tGD “swapped” version targeting *yellow* (gRNAs-Red) and *white* (Cas9-Green). Inheritance rates using *vasa* promoter with Cas9 inherited from F_0 males.
6. **Supp. Fig. 3(A) - tGD(y,w) - Raw data:** tGD targeting *yellow* (Cas9-Red) and *white* (gRNA-Green). Inheritance rates using *nanos* promoter with Cas9 inherited from F_0 males.
7. **Supp. Fig. 3(B) - tGD(y,w) - Raw data:** tGD targeting *yellow* (Cas9-Red) and *white* (gRNA-Green). Inheritance rates using *nanos* promoter with Cas9 inherited from F_0 females.
8. **Supp. Fig. 3(C) - tGD(y,w) - Raw data:** tGD targeting *yellow* (Cas9-Red) and *white* (gRNA-Green). Inheritance rates using *nanos* promoter with Cas9 and gRNA transgenes inherited from F_0 males.
9. **Supp. Fig. 3(D) - tGD(y,w) - Raw data:** tGD targeting *yellow* (Cas9-Red) and *white* (gRNA-Green). Inheritance rates using *nanos* promoter with Cas9 and gRNA transgenes inherited from F_0 females.
10. **St: Fig.1B,C vs. Fig.2A,E (Cas9 in yellow) - Statistical analysis**: tGD comparison targeting *yellow* (Cas9-Red) between tGD(y,e) and tGD(y,w). The Cas9-DsRed element is inserted in the *yellow* gene in both cases but the *y*-gRNA source is located at the *ebony* and *white* genes, respectively.
11. **St: Fig.2A,E vs. Fig.S2** - **Statistical analysis**: tGD(y,w) with the Red-Cas9 and gRNAs-Green inserted in *yellow* and *white* respectively, compared to the tGD(w,y) or “switched version” where the gRNAs-Red and Cas9-Green where instead inserted at the *yellow* and *white* locus, respectively.
12. **St: Fig.2A,E vs. Fig.S3 - Statistical analysis**: Inheritance differences between the Cas9-Red and gRNAs-Green in the tGD(y,w) driven by *vasa* compared to the tGD(y,w) driven by *nanos*.

Title: Supplementary Data 3 - Phenotypic analysis of the F_2 progeny of drug-inducible tGD(y,w) crosses performed.

Description: Raw counting data of the F_2 progeny phenotypic scoring indicating females and males recovered. Red marker (**DsRed+**), green marker (**GFP+**), both fluorophores (**both**) or no fluorescence (**none**) were scored in order to track Cas9 (red marker) and gRNAs (green marker) transgenes. Transgene inheritance rates in the F_2 progeny for each specific tube (marked as **cross#** in the table) were calculated by combining data from males and females. Average inheritance for both markers and the standard deviation are calculated as well. Both spCas9 and DD2-spCas9 were tested in regular food (**TMP-**) and under 80μM Trimethoprim exposure (**TMP+**). Data is divided in the following file tabs:

1. **Supp. Fig. 6B (SpCas9) - tGD(y,w) - Raw data.** tGD targeting *yellow* (Cas9-Red) and *white* (gRNA-Green) using *vasa* promoter and SpCas9.
2. **Supp. Fig. 6B (DD2-SpCas9) - tGD(y,w) - Raw data.** Drug-inducible tGD (DD2-SpCas9) targeting *yellow* (Cas9-Red) and *white* (gRNA-Green) using *vasa* promoter. The eye phenotype of F_2 flies were scored to distinguish wild-type, red eye (**w+**), white eye (**w-**) or mosaic eye (**w+/w-**) in both females and males. We also scored flies for yellow body color (**yellow-**) or wild-type body color (**yellow+**).
3. **Supp. Fig. 6C - tGD(y,w) - Raw data.** Daily analysis of gene drive activity in the adult germline using the drug-inducible tGD (DD2-SpCas9) targeting *yellow* (Cas9-Red) and *white* (gRNA-Green) driven by *vasa* promoter. The eye phenotype of F_2 flies were scored to distinguish wild-type, red eye (**w+**), white eye (**w-**) or mosaic eye (**w+/w-**) in both females and males. We also scored flies for yellow body color (**yellow-**) or wild-type body color (**yellow+**).
4. **Supp. Fig. 6C - Summary - tGD(y,w) - Raw data.** Summary of the gene drive activation in the adult germline over 10 consecutive days. The eye phenotype of F_2 flies were scored to distinguish wild-type, red eye (**w+**), white eye (**w-**) or mosaic eye (**w+/w-**) in both females and males. We also scored flies for yellow body color (**yellow-**) or wild-type body color (**yellow+**).

Title: Supplementary Data 4 - Phenotypic analysis of the F_2 progeny of our gene drive elements with impaired homology arms.

Description: Raw counting data of the F_2 progeny phenotypic scoring indicating females and males recovered. Red marker (**DsRed+**), green marker (**GFP+**), both fluorophores (**both**) or no fluorescence (**none**) were scored in order to track Cas9 (red marker) and gRNAs (green marker) transgenes. Transgene inheritance rates in the F_2 progeny for each specific tube (marked as **cross#** in the table) were calculated by combining data from males and females. Average inheritance for both markers and the standard deviation are calculated as well. The eye color under regular brightfield conditions of each single fly was also scored to distinguish red eye (**w+**), white eye (**w-**) or mosaic eye (**w+/w-**) in both females and males. Data is divided in the following file tabs:

1. **Fig. 5A (both sides) - tGD(y,w) - Raw data.** tGD targeting *yellow* (Cas9-Red) and *white* (gRNA-Green) with the gRNA transgene lacking 20 nucleotides on both sides.
2. **Fig. 5A (PAM-proximal) - tGD(y,w) - Raw data.** tGD targeting *yellow* (Cas9-Red) and *white* (gRNA-Green) with the gRNA transgene lacking 20 nucleotides in the PAM-proximal side.
3. **Fig. 5A (PAM-distal) - tGD(y,w) - Raw data.** tGD targeting *yellow* (Cas9-Red) and *white* (gRNA-Green) with the gRNA transgene lacking 20 nucleotides in the PAM-distal side.
4. **St: Fig.2A,E vs. Fig.5A - Statistical analysis**: tGD(y,w) with perfect homology compared to the tGD(y,w) with the "truncated" PAM-proximal and PAM-distal gRNAs-Green missing specific homology.

5. **St: Fig.5C-E - Statistics analysis**: Percentage of dots under the diagonal in the tGD(y,w) with perfect homology in the gRNAs-Green cassette compared to the tGD(y,w) “truncated” PAM-proximal and PAM-distal gRNAs-Green cassettes missing specific homology.
