# Supplementary material for: A transcomplementing gene drive provides a flexible platform for laboratory investigation and potential field deployment
Source: Nat Commun. 2020 Jan 17;11:352. doi: 10.1038/s41467-019-13977-7 (PMC6969112; doi:10.1038/s41467-019-13977-7)
Supplement: Supplementary file 8 — Reporting Summary [file 41467_2019_13977_MOESM8_ESM.pdf]

nature research

Corresponding author(s): Valentino Gantz

Last updated by author(s): Dec 7, 2019

# Reporting Summary

Nature Research wishes to improve the reproducibility of the work that we publish. This form provides structure for consistency and transparency in reporting. For further information on Nature Research policies, see Authors & Referees and the Editorial Policy Checklist .

## Statistics

For all statistical analyses, confirm that the following items are present in the figure legend, table legend, main text, or Methods section.

| n/a | Confirmed | |
|---|---|---|
| ☐ | ☒ | The exact sample size (*n*) for each experimental group/condition, given as a discrete number and unit of measurement |
| ☐ | ☒ | A statement on whether measurements were taken from distinct samples or whether the same sample was measured repeatedly |
| ☐ | ☒ | The statistical test(s) used AND whether they are one- or two-sided<br>*Only common tests should be described solely by name; describe more complex techniques in the Methods section.* |
| ☒ | ☐ | A description of all covariates tested |
| ☒ | ☐ | A description of any assumptions or corrections, such as tests of normality and adjustment for multiple comparisons |
| ☐ | ☒ | A full description of the statistical parameters including central tendency (e.g. means) or other basic estimates (e.g. regression coefficient) AND variation (e.g. standard deviation) or associated estimates of uncertainty (e.g. confidence intervals) |
| ☐ | ☒ | For null hypothesis testing, the test statistic (e.g. *F*, *t*, *r*) with confidence intervals, effect sizes, degrees of freedom and *P* value noted<br>*Give P values as exact values whenever suitable.* |
| ☒ | ☐ | For Bayesian analysis, information on the choice of priors and Markov chain Monte Carlo settings |
| ☒ | ☐ | For hierarchical and complex designs, identification of the appropriate level for tests and full reporting of outcomes |
| ☒ | ☐ | Estimates of effect sizes (e.g. Cohen's *d*, Pearson's *r*), indicating how they were calculated |

*Our web collection on statistics for biologists contains articles on many of the points above.*

## Software and code

Policy information about availability of computer code

| | |
|---|---|
| Data collection | Microsoft Excel 2011, Microsoft Excel 16.16.6, and Google Sheets were used for data collection. |
| Data analysis | Graphpad Prism 7 & 8 and Adobe Illustrator Creative Cloud were used for data analysis and display.<br><br>The code for running the simulation of the mathematical modeling presented in the manuscript is freely available from the MGDrivE GitHub repository [https://github.com/MarshallLab/MGDrivE], and the package can be installed on R through CRAN [https://cran.r-project.org/web/packages/MGDrivE/]. The inheritance cubes used in the simulations are the “cubeTGD” and “cubeTGDX” variants of the codebase. |

For manuscripts utilizing custom algorithms or software that are central to the research but not yet described in published literature, software must be made available to editors/reviewers. We strongly encourage code deposition in a community repository (e.g. GitHub). See the Nature Research guidelines for submitting code & software for further information.

## Data

Policy information about availability of data

All manuscripts must include a data availability statement. This statement should provide the following information, where applicable:

- Accession codes, unique identifiers, or web links for publicly available datasets
- A list of figures that have associated raw data
- A description of any restrictions on data availability

The sequence of all plasmid constructs generated in this manuscript have been deposited into the GenBank database with accession codes: MN551085, MN551086, MN551087, MN551088, MN551089, MN551090, MN551091, MN551092, MN551093, MN551094. In this study we accessed the Addgene plasmid # 49411 ; http://n2t.net/addgene:49411 ; RRID:Addgene_49411. All raw phenotypical scoring data collected is reported in the Supplementary Data 1-4 files in Microsoft Excel format (.xlsx) available as Source Data file. All other data is available from the authors.

# Field-specific reporting

Please select the one below that is the best fit for your research. If you are not sure, read the appropriate sections before making your selection.

☒ Life sciences ☐ Behavioural & social sciences ☐ Ecological, evolutionary & environmental sciences

For a reference copy of the document with all sections, see nature.com/documents/nr-reporting-summary-flat.pdf

# Life sciences study design

All studies must disclose on these points even when the disclosure is negative.

| | |
|---|---|
| Sample size | In our previous experience of similar analysis of gene drive effect using single fly pair crosses a number size of >8 is usually representative, describing the overall behavior. For each of our experimental condition we have collected between 11-89 different samples. |
| Data exclusions | All raw data provided was included in the figures. Fruit fly crosses with no progeny due to contamination or other causes, were removed from the analysis and are not reported in the raw data tables. |
| Replication | All the drive inheritance experiments performed include multiple independent technical replicates plotted as individual data points. Additionally, for the tGD proof-of-principle we provide evidence that our observations are reproducible as an independent experiments were performed using different reagents (yellow/ebony-tGD, yellow/white-tGD and white/yellow-tGD). |
| Randomization | F1 female flies were randomly collected from different F0 crosses to perform F1 crosses. |
| Blinding | For all the experiments performed we have analyzed fluorescence presence in the eyes of fruit flies. This type of scoring does not need the investigators to be blind as the evaluation of the phenotype is presence or absence of the fluorescent marker and there is no much room for interpretation that could be subjective. |

# Reporting for specific materials, systems and methods

We require information from authors about some types of materials, experimental systems and methods used in many studies. Here, indicate whether each material, system or method listed is relevant to your study. If you are not sure if a list item applies to your research, read the appropriate section before selecting a response.

## Materials & experimental systems

| n/a | Involved in the study |
|---|---|
| ☒ | ☐ Antibodies |
| ☒ | ☐ Eukaryotic cell lines |
| ☒ | ☐ Palaeontology |
| ☐ | ☒ Animals and other organisms |
| ☒ | ☐ Human research participants |
| ☒ | ☐ Clinical data |

## Methods

| n/a | Involved in the study |
|---|---|
| ☒ | ☐ ChIP-seq |
| ☒ | ☐ Flow cytometry |
| ☒ | ☐ MRI-based neuroimaging |

## Animals and other organisms

Policy information about studies involving animals; ARRIVE guidelines recommended for reporting animal research

| | |
|---|---|
| Laboratory animals | All Drosophila melonogaster transgenic animals were generated or assembled in Oregon-R genetic background. |
| Wild animals | Study did not involve wild animals. |
| Field-collected samples | Study did not involve field-collected samples. |
| Ethics oversight | Drosophila melonogaster is an invertebrate and is exempt from IACUC oversight. |

Note that full information on the approval of the study protocol must also be provided in the manuscript.
